# The Eastern Quebec Telepathology Network: a support to the improvement to the public health care system

**DOI:** 10.1186/1746-1596-8-S1-S8

**Published:** 2013-09-30

**Authors:** Bernard Têtu, Marie-Pierre Gagnon, Geneviève Roch, Jean-Paul Fortin

**Affiliations:** 1 Professor of pathology and Medical Director of The Eastern Quebec Telepathology Network; Departement of Pathology, Laval University, Québec, Canada; 2 Associate professor, Faculty of Nursing Sciences, Laval University, Québec, Canada; 3 Adjunct professor, Faculty of Nursing Sciences, Laval University, Québec, Canada; 4 Professor, Department of Social and Preventive Medicine, Laval University, Québec, Canada

## Summary

The Eastern Quebec Telepathology Network is aimed at providing uniform diagnostic telepathology services in a huge territory with a low population density. It has been designed to provide intraoperative consultations (frozen sections) in smaller community hospitals and to allow pathologists working alone to rapidly obtain a second opinion. This study provides an interim evaluation of the benefits of the network. The network involves 24 sites, of which seven are devoid of a pathology laboratory, three have no pathologist and six have one pathologist on site. Since the beginning of the implementation, the coverage in pathology in this territory was improved: 1) telepathology allowed pathologists to work outside of their office and to provide surgeons with faster diagnoses of urgent biopsies; 2) expected interruptions of the intraoperative consultation coverage were avoided ; 3) intraoperative consultations were provided to surgeons in hospitals devoid of pathology laboratory; 4) expert opinions were obtained with reduced isolation for pathologists working alone and improved turn-around time; 5) merging of smaller laboratories resulted in a more stable pathology coverage and an attractive effect on the recruitment of young pathologists; 6) videoconferencing and macroscopy station allowed real-time communication between a pathologist and the remote technician for macroscopic description; and 7) several technical procedures were standardized (staining, sectioning, reporting). In conclusion, the Eastern Quebec Telepathology Network was designed to improve medical care to patients. In a short period of time, an improvement of the organization of health cares and of the delivery of services is already apparent.

## Background

**Figure 1:**
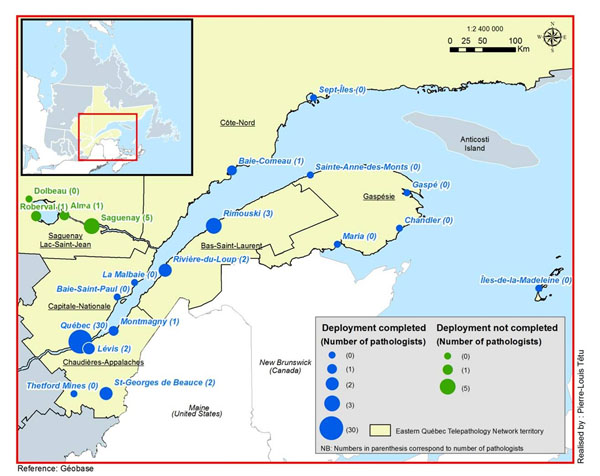
Territory covered by the Eastern Quebec Telepathology Network. (Adapted from figure 1 published in Médecine/Science, 2012; 28:993-9 and used with permission from the editor).

Canada is a huge country with a low population density. The province of Quebec is the largest but the second most populated province with its 7,957,600 inhabitants. The territory covered by the Eastern Quebec Telepathology Network spans over 452,600 km^2^ in which 1,7 million inhabitants live (figure 1). In certain areas, the density is as low as 0.4 inhabitants/km^2^.

In 2004, a telepathology project team was asked to perform a survey exploring and evaluating specific needs for telepathology in Eastern Quebec. This survey revealed that, due to the lack of consistent pathology coverage in several smaller community hospitals, certain surgeries were required to be postponed, several patients were transferred to regional hospitals and two-step surgeries had to be performed when an intraoperative consultation was needed and no pathologist was available on site. This situation also proved to be a major limitation for the recruitment of young surgeons trained in centers where access to an expert pathologist is never an issue. Furthermore, it was clear that younger pathologists in early practice felt insecure and were often reluctant to work alone because of the difficulty in rapidly obtaining a second opinion from a colleague. Practicing pathologists complained that they could hardly be absent without disturbing the surgical unit. Recent literature indicates that telepathology may be instrumental in reforming the health care system and improving the quality of care [1]. In this context, the Eastern Quebec Telepathology Network has been created and was aimed at improving pathology coverage in remote communities by sharing regional expertise to ensure consistent intraoperative consultation and access to expert opinions.

The Quebec Ministry of Health and Canada Health Infoway agreed to financially support this innovative initiative with the objective of providing the population with uniform pathology services. Deployment began in January 2010 and two years later, the implementation and training of technical and medical staff are nearly complete and the network is functional. Recently, we identified a number of challenges which, while not threatening the deployment, nevertheless required a strategy to insure that the pace of the implementation was maintained [2]. The objective of this paper is to share our experience and provide an interim evaluation of the benefits of the network as its implementation is underway.

## Methods

Each hospital of the network is equipped with a whole slide scanner (Nanozoomers, Hamamatsu Photonics, *Shizuoka Prefecture*, *Japan*), a macroscopy station (PathStand 40, Diagnostic Instruments, *Sterling Height*, *USA*) and two videoconferencing devices (PCS-XG80DS Codec, Sony, *Minato*, *Tokyo*, *Japan*) equipped with a drawing tablet (Bamboo CTE-450K, WACOM, *Otone*, *Saitama*, *Japan*). Those equipments were obtained from Olympus Canada.Inc (*Markham*, Canada). The viewer and case management and collaboration system selected is mScope (Aurora Interactive Ltd., *Montreal*, *Canada*). All hospitals in the province of Quebec are linked together by a private, dedicated information network which ensures confidentiality and avoids use of the Internet (RITM: réseau intégré de télécommunications multimédias). The project has been implemented in a total of 24 sites, of which seven are devoid of a pathology laboratory. Of the remaining 17 sites, three have no pathologist, six have one pathologist and eight have two or more pathologists (figure 1).

Each of the 24 sites was visited by the project team prior to the deployment. The project team met the local medical (surgeons and pathologists), technical (informatics and biomedical) and administrative teams in order to better understand their needs, provide project information and identify potential locations for the equipment. A second visit was performed one year later in each center to gather invaluable information regarding the benefits and challenges met in the course of implementation.

## Results and discussion

The Eastern Quebec Telepathology Network is currently the most ambitious telepathology project in Canada [3] and ranks among the most important in the world in terms of both the number of sites and geographic coverage. Essentially, the activities include remote intraoperative consultations (frozen sections), expert opinions between pathologists, remote assistance to macroscopic description, rapid return of immunohistochemistry and primary diagnosis on paraffin sections, including the request by surgeons of rapid diagnoses for urgent medical decision. Updated data on the use of telepathology in the Network for the period of September 2010 to January 2012 is provided in table 1.

**Table 1:** Analyses performed by telepathology between September 2010 and June 2012.

Analysis	Number of slides scanned
Primary diagnosis, including urgent interpretation	7108

Intraoperative consultations (frozen sections)	473

Expert opinions between pathologists	505

Assistance to macroscopic description	166

Immunohistochemistry	149

Basically, thus far, we have found that this network encouraged more collaboration between surgeons, technologists and pathologists in a region and resulted in a better overall regional organization of medical care. More specifically, the main benefits identified to date have been:

1) Most slides were scanned for primary diagnosis (7108 slides scanned). Indeed, telepathology allowed pathologists to work outside of their office and to provide surgeons with faster diagnoses of urgent biopsies for faster planning of patients care, despite the lack of an on-site pathologist;

2) Expected interruptions of the intraoperative consultation coverage were avoided (473 slides scanned) with maintenance of local surgical and pathology technical activities. The process was intended to allow pathologists and surgeons to follow the same steps as if they were working in a single hospital. Our experience to date [2] shows that the time required is competitive with the current situation in a single hospital and compares favorably with data from the literature [4];

3) Intraoperative consultations were provided to oncologic surgeons in hospitals devoid of pathology laboratory. This is an innovative application of this technology and required proper training to technologists with limited training in histology;

4) Many expert opinions were obtained (505 slides scanned) and immunohistochemical analyses were returned more rapidly (149 slides scanned), supporting pathologists working alone and resulting in a significant reduction of the turn-around time. The system allows a referring pathologist to request an opinion from any other pathologist in the network. Indeed, it is estimated that 10 to 20% of oncologic cases must be validated by more than one pathologist [5] and certain quality assurance programs require that 10% of cases be reviewed by more than one pathologist [6]. Current literature shows that expert opinion is an area with enormous growth potential for telepathology [7];

5) The merging of smaller laboratories in a sub-region was encouraged with the result of a more stable intraoperative consultation coverage and an attractive effect on the recruitment of young pathologists in this sub-region. Prior studies by two of us (MPG, JPF) showed that telemedicine technologies may help to attract physicians to and retain them in remote regions by contributing to better working conditions [8,9];

6) The videoconferencing and macroscopy station allowed for real-time communication between a pathologist and the remote technician or pathologist assistant for macroscopic description of specimens (166 sessions) resulting in a decreased need for specimen transportation;

7) Several technical procedures such as staining, sectioning and reporting had to be standardized in different institutions working together. There is increasing literature suggesting that technical improvements and standardization enhance the quality of images by telepathology [10].

## Conclusion

The Eastern Quebec Telepathology Network is successful and improves medical care and of the delivery of services to patients in this region. In the course of implementation, the objectives that we had settled were not only met but were also exceeded. The learning experience of such a project may be helpful to any organization intending to implement a public health / patient-oriented telepathology network.

## Competing interests

The authors declare that they have no competing interests.

## Authors’ contributions

All authors contributed to the development, writing and approval of the final manuscript.
